# K_2p_3.1 protein is expressed as a transmural gradient across the rat left ventricular free wall

**DOI:** 10.1111/jce.13805

**Published:** 2018-12-28

**Authors:** Sandra A. Jones, Richard D. Walton, Michael Morton, Matthew K. Lancaster

**Affiliations:** ^1^ Department of Biomedical Sciences, Department Faculty of Health Sciences University of Hull Hull UK; ^2^ Centre de Recherche Cardio‐Thoracique de Bordeaux U1045, Université de Bordeaux Bordeaux France; ^3^ Inserm, Centre de Recherche Cardio‐Thoracique de Bordeaux Bordeaux France; ^4^ L'Institut de Rythmologie et Modélisation Cardiaque LIRYC, Fondation Bordeaux Université Bordeaux France; ^5^ ApconiX Ltd. 3F68 Alderley Edge Alderley Park UK; ^6^ Faculty of Biological Sciences, School of Biomedical Sciences, University of Leeds Leeds UK

**Keywords:** adrenergic tone, heterogeneity, ischemia, K2p3.1, proarrhythmia, TASK‐1, transmural gradient

## Abstract

**Introduction:**

K_2p_3.1, also known as TASK‐1, is a twin‐pore acid‐sensitive repolarizing K^+^ channel, responsible for a background potassium current that significantly contributes to setting the resting membrane potential of cardiac myocytes. Inhibition of I_K2p3.1_ alters cardiac repolarization and is proarrhythmogenic. In this study, we have examined the expression of K_2p_3.1 and function of this channel in tissue and myocytes from across the left ventricular free wall.

**Methods and Results:**

Using fluorescence immunocytochemistry, the expression of K_2p_3.1 protein in myocytes from the subendocardial region was found to be twice (205% ± 13.5%) that found in myocytes from the subepicardial region of the left ventricle (100% ± 5.3%). The left ventricular free wall exhibited a marked transmural gradient of K_2p_3.1 protein expression. Western blot analysis confirmed significantly higher K_2p_3.1 protein expression in subendocardial tissue (156% ± 2.5%) than subepicardial tissue (100% ± 5.0%). However, there was no difference in K_2p_3.1 messenger RNA expression. Whole‐cell patch clamp identified I_K2p3.1_ current density to be significantly greater in myocytes isolated from the subendocardium (7.66 ± 0.53 pA/pF) compared with those from the subepicardium (3.47 ± 0.74 pA/pF).

**Conclusions:**

This is the first study to identify a transmural gradient of K_2p_3.1 in the left ventricle. This gradient has implications for understanding ventricular arrhythmogenesis under conditions of ischemia but also in response to other modulatory factors, such as adrenergic stimulation and the presence of anesthetics that inhibits or activates this channel.

## 1 INTRODUCTION

K_2p_3.1 (TWIK‐related acid‐sensitive K^+^ channel; GenBank accession number AF031384, rattus) alternatively known as “TASK‐1,” is a member of the twin‐pore K^+^ channel family, comprising four transmembrane segments and two pore‐forming domains with intracellular termini. Duprat et al^1^ first described the properties of K_2p_3.1 as a background conductance channel with instantaneous activation and a current‐voltage relationship that indicated an openly rectifying, K^+^ selective, pore function. The principal modifier of K_2p_3.1 channel functionality is considered to be external pH with the channel showing a dynamic functional response within the normal physiological range, with maximal activation at pH more than 7.8 but complete inhibition at pH values less than 6.4.^1^, ^2^, ^3^ The pK of the channel is ~7.3 with a Hill coefficient of ~1.6, thus showing a steep sensitivity to pH across this key physiological and pathophysiological range.^1^ Channel functionality of K_2p_3.1 can also be modulated by several other factors: inhibition occurs during hypoxia, in response to α adrenergic^4^ and endothelin‐1 receptor stimulation^5^ and in the presence of the endocannabinoid anandamide (or its stable analog methanandamide).^6^, ^7^ It also shows phosphorylation‐dependent inhibition by protein kinase C^7^ and inhibition by platelet activating factor.^8^ In contrast, activation of K_2p_3.1 channels occurs in response to the key inhalational anesthetics halothane, sevoflurane, and isoflurane.^4^, ^9^ The broad spectrum of intrinsic and extrinsic factors that dynamically modulate this channel make this an important target of interest for understanding the response of the cardiac action potential to numerous stressors and conditions.

K_2p_3.1 protein has been demonstrated to be present in both the atria and ventricles of the heart.^2^, ^10^, ^11^ In myocytes derived from human atria samples, I_K2p3.1_ has been shown to contribute significantly to repolarization of the myocyte and constitutes approximately 40% of the background potassium current.^12^ Inhibition or complete genetic knockout of K_2p_3.1 channels in the mouse heart causes a significant prolongation of the ventricular action potential duration (APD) (by approximately 17%) and an increase in QTc and QRS intervals (by approximately 30% each).^13^, ^14^ In ventricular myocytes, similarly to atrial myocytes, I_K2p3.1_ contributes to repolarization and shortening of the APD.^15^, ^16^ This impact has been demonstrated to vary between cells with Putzke et al,^16^ suggesting this may be due to variation in the specific origin of the myocytes in such pooled cell preparations and their associated action potential characteristics. Within this study, they were able to model the impact of current through K_2p_3.1 using the “Pandit” mathematical model of the rat ventricular subepicardial myocyte,^17^ identifying a predicted significant prolongation of the APD if this current were inhibited in myocytes from this region.^16^ No previous investigations have, however, directly investigated potential heterogeneity of I_K2p3.1_ expression within the ventricular wall. Such heterogeneity would have implications for understanding the response of the heart to the numerous changes altering the activity of this channel and its associated current. Our hypothesis was, therefore, that a heterogeneous expression pattern of K_2p_3.1 and associated current exists within the left ventricular free wall. Such a difference would have the potential to impact the dispersion of repolarization across the wall of the heart in the event of changes in pH and many other physiological and pathological signals, predisposing to arrhythmias.

## 2 MATERIALS AND METHODS

### 2.1 Acquisition of tissue and single myocytes

Male Wistar rats aged 5 to 6 months were humanely killed via concussion followed by cervical dislocation. All work was performed in accordance with the Animals (Scientific Procedures) Act 1986 and approved by local ethics committees. Each heart was dissected to remove the left ventricular free wall. The cross‐section of the ventricular wall for immunocytochemistry was oriented in cryomedia and snap frozen in isopentane cooled by liquid nitrogen. For Western blot analysis and messenger RNA (mRNA) analysis the left ventricular free wall was dissected into the subepicardial and subendocardial layers (~30% of the total wall thickness from each surface), the midlayer and outer endothelium layers were discarded; regional tissue samples were frozen in liquid nitrogen and stored at −80°C until used.

The preparation of subepicardial and subendocardial single myocytes used a variation of a method previously described by Harrison et al.^18^ Briefly, each heart was perfused via the aorta with a 4‐(2‐hydroxyethyl)‐1‐piperazineethanesulfonic acid (HEPES)‐buffered saline solution containing (mM): NaCl, 130; KCl, 5.4; MgCl_2_, 1.4; NaH_2_PO_4_, 0.4; HEPES, 5; glucose, 10; creatine, 10; taurine, 20; adjusted to pH 7.4 using NaOH and bubbled with 100% O_2_. All solutions were maintained at 35°C. Initially, the perfusate also contained 0.75 mM CaCl_2_ but once the heart was clear of blood (1 minute of perfusion) the perfusate was changed to a 0 mM Ca^2+^ version of the saline solution also containing 0.1 mM ethylene glycol‐bis(β‐aminoethyl ether)‐*N*,*N*,*N*′,*N*′‐tetraacetic acid (EGTA). After 4 minutes perfusion the solution was supplemented with Type 1 collagenase (Worthington Biochemical Corp., Lakewood, NJ), 0.8 mg/mL and protease type XIV (Sigma‐Aldrich, Dorset, UK) 0.1 U/mL for 6 minutes of perfusion. The heart was then removed from the aortic cannula, the left ventricular free wall removed and was dissected into the subepicardial and subendocardial layers. The isolated regions were agitated in the basic saline solution supplemented with 10% horse serum to disperse single myocytes. The resultant suspension of cells was centrifuged at 300 rpm to form a pellet and resuspended in a fresh solution containing 0.2 mM Ca^2+^ for storage until use.

### 2.2 Analysis of protein expression

For immunofluorescence, as previously described by Jones et al,^10^ single myocytes and tissue cryosections of 10 µm thickness were fixed using 4% paraformaldehyde in phosphate‐buffered saline (PBS) for 20 minutes, followed by washing. Myocytes and tissue were immersed in Triton X‐100, 0.05% in PBS for 20 minutes, followed by further washing. After 1 hour in a blocking solution containing 20% horse serum and 0.1% bovine serum albumin an anti‐K_2p_3.1 primary antibody (rabbit polyclonal; Alomone, Jerusalem, Israel) was applied at 1:200 diluted in blocking solution and incubated overnight at 4°C. After washing to remove excess antibody, Alexa Fluor 488 secondary antibody (Molecular Probes, Eugene, OR) was applied to the sections at 1:1000 for 1 hour at room temperature and again washed. All myocytes and tissue sections were mounted in Vectashield (Vector Labs, Peterborough, UK). The intensity of the fluorescent antibody complex with K_2p_3.1 protein was measured by laser scanning microscopy using an LSM5 Pascal Zeiss confocal microscope equipped with a ×40 objective and set to collect light from a 1 µm optical slice (Zeiss, Oberkochen, Germany). Image stacks of the entirety of individual myocytes were collected for analysis.

Quantification of K_2p_3.1 protein by Western blot analysis was carried out as previously described by Jones et al.^10^ In brief, tissue samples were crushed under liquid nitrogen, suspended in protease inhibiting buffer and centrifuged at 12 000 rpm for 10 minutes at 4°C (Eppendorf, Stevenage, UK). The pellet was discarded and the cell lysate protein content determined using a bicinchoninic acid assay (Pierce, Waltham, MA). Samples (50 µg protein/lane) were separated by 10% sodium dodecyl sulphate polyacrylamide gel electrophoresis (SDS‐PAGE) electrophoresis and transferred to nitrocellulose membranes by semidry discontinuous blotting (Pharmacia, Buckinghamshire, UK). Nonspecific binding sites on the membrane were blocked by incubation overnight at 4°C in SuperBlock (Pierce). Anti‐K_2p_3.1 was applied (1:1000) in blocking solution overnight at 4°C. Membranes were washed in Tween 20 (0.05%)—PBS. The membrane was incubated with a secondary anti‐rabbit antibody conjugated to horseradish peroxidase (HRP) (DAKO, Santa Clara, CA) for 2 hours in blocking solution, then washed. The membrane‐bound complex of K_2p_3.1 protein and antibody were detected as a band at 130 kDa. by the ECL system (Pierce) using hyperfilm (GE Healthcare, Amersham, UK). The procedure was repeated on the same membrane using an anti‐desmin antibody (Sigma‐Aldrich, Dorset, UK) and secondary anti‐mouse antibody conjugated to HRP (DAKO). The antibody complex was identified as a specific band at 53 kDa and the band integrated signal per sample used to verify equal protein loading of tissue.

### 2.3 Detection of mRNA expression

Total RNA was extracted from subendocardial and subepicardial layers using the Gen Elute extraction kit (Sigma‐Aldrich). Reverse transcription was performed on 1 µg of total RNA per sample. Polymerase chain reaction (PCR) was conducted on the ensuing reverse‐transcribed material with 10 pmol of each primer, 20 mM Tris pH 8.8, 10 mM KCl, 10 mM (NH4)_2_SO_4_, 2 mM MgSO4, 0.1% Triton X‐100, 100 µg/mL bovine serum albumin and 200 nM dNTPs. Using a Geneamp 2400 cycler (PE Biosystems, Waltham, MA) a “hot‐start” was initiated and 2.5 U Taq DNA polymerase added: there were 30 cycles of PCR (94°C 30 seconds; 60°C 45 seconds; 72°C 30 seconds). The two sets of specific primer pairs used were rat K2p3.1 5′‐ACGATGAAGCGGCAGAATGTG‐3′ (sense) with 5′‐ACGAAACCGATGAGCCATG‐3′ (antisense) and β‐actin 5′‐TTGTAACCAACTGGGACGATATGGG‐3′ (sense) with 5′‐GATCTTGATCTTCATGGTGCTAGG‐3′ (antisense). All conditions, solutions, and primers are as previously described by Jones et al.^10^

### 2.4 Electrophysiological recordings

The method for recording K2p3.1 currents was adapted from Besana et al.^19^ Subepicardial or subendocardial myocytes were initially superfused at room temperature with a Tyrode's buffer, containing (mM): NaCl, 140; KCl, 5.4; MgCl_2_, 1; glucose, 10; HEPES, 5; CaCl_2_, 1; adjusted to pH 7.4 using NaOH. Whole‐cell patch clamp was performed using glass microelectrodes with a tip resistance of 4 to 6 MΩ filled with a pipette solution containing (mM): l‐aspartic acid, 146; KOH, 130; NaCl, 10; EGTA, 5; HEPES, 10; MgATP, 2; CaCl_2_, 2; adjusted to pH 7.2 using KOH. K_2p_3.1 current was recorded in modified Tyrode's solution containing (mM): NaCl, 100; KCl, 50; MgCl_2_, 1; HEPES, 5; glucose, 10; tetraethylammonium chloride, 1; CsCl, 5; CaCl_2_, 1, adjusted to pH 7.4 using NaOH. Cells were held at −10 mV in the presence of the high K^+^ solution and allowed to equilibrate for 6 minutes before measuring currents.

R(^+^)‐Methanandamide (Sigma‐Aldrich), a selective inhibitor of the K_2p_3.1 channel, was dissolved in ethanol (5 mg/mL), and further diluted to a final working concentration of 10 µM. To selectively identify current through K_2p_3.1 channels, a 6 second voltage ramp protocol from −50 to 30 mV was performed in the presence and absence of the channel inhibitor with the difference current determined by subtraction. Cell capacitance was assessed using 10 mV hyperpolarizing pulses from a holding potential of −80 mV. Recordings were made using an Axopatch 200B amplifier (Molecular Devices, San Jose, CA) with digital/analog conversion via a Digidata 1322 interface (Molecular Devices). During K_2p_3.1 current recordings whole‐cell capacitance was compensated and series‐resistance compensation was typically 70% to 75%. Data were acquired at 5 kHz with a low pass Bessel filter of 1 kHz.

### 2.5 Analysis of data

Immunofluorescent images of sections and single myocytes were taken using identical settings. Fluorescence intensity profiles of 10 microns width were taken perpendicular to the epicardial and endocardial surfaces across the ventricular wall. Average intensities of fluorescence were calculated for each 20% of the distance across the total transmural distance of the left ventricular free wall from the subepicardium to the subendocardium. The collective data for each interval were normalized to the fluorescence intensity of the first subepicardial interval (0%‐20% of the distance across the wall).

Electrophysiological data were analyzed using Clampfit 9 (Molecular Devices). Records were not corrected for the liquid junctional potential that was measured to be −9.8 mV. Peak current density comparisons were made at 30 mV. Current density measurements were expressed relative to the whole‐cell capacitance, which was determined as the integral of the whole‐cell capacitance transient. I_K2p3.1_ was taken as the methanandamide‐sensitive current.

### 2.6 Statistical analysis

Data are expressed as means ± standard error of the mean (SEM) and *n* = number in the group. A minimum of six animals were used per experimental grouping (*n*), corresponding to protein/tissue samples or electrophysiology results as appropriate. For the tissue cross‐sectional analysis, 6 to 8 sections were taken at distributed points down the left ventricular wall as obtained from seven animals. Statistical assessment was performed by two‐way Student's *t* test or one‐way analysis of variance with Holm‐Sidak post hoc comparisons, as appropriate. Significance was determined if *P* < 0.05.

## 3 RESULTS

### 3.1 K_2p_3.1 protein expression in single myocytes isolated from subepicardial and subendocardial layers of the rat left ventricle

**Figure 1:**
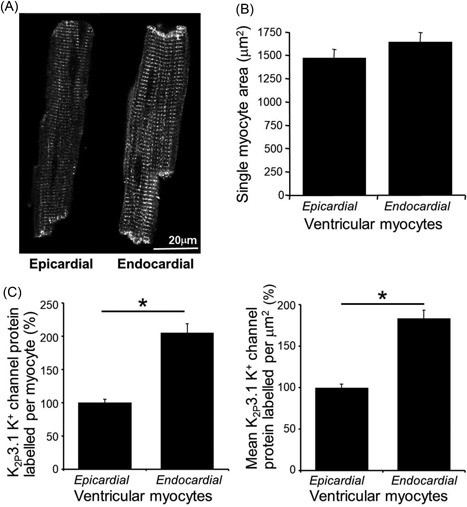
Single epicardial and endocardial myocytes immunofluorescent label of K_2_p3.1 protein. A, Illustrative example high‐resolution images of K_2p_3.1 protein labelling in single myocytes from the subepicardial (left) and subendocardial (right) regions of the left ventricle. B, Single myocyte cross‐sectional area for cells from each region. C, Mean total immunofluorescence of labelled K_2p_3.1 protein per myocyte. D, The mean density of immunofluorescence of labelled K_2p_3.1 protein (each panel shows means ± SEM; *n* = 92 cells per group; bars denote significant differences by *t* test; *P < 0.001)

Specificity of the antibody to *C*‐terminal K_2p_3.1 protein for use in immunofluorescence with confocal microscopy was previously determined by Jones et al.^10^ In the present study, myocytes isolated from subepicardial tissue exhibited visibly less labelled K_2p_3.1 protein than those isolated from the subendocardial tissue; both cell types exhibited a similar pattern of strong staining at the intercalated disc with transversely oriented fluorescence striations consistent with t‐tubules across the myocyte (Figure 1A). The individual projected area of cells from each region was determined and did not differ significantly between regions (Figure 1B; *n* = 92). At mid‐cell depth, a single optical slice was used to assess the density of channel expression. Myocytes isolated from the subendocardial region demonstrated a two‐fold greater integrated fluorescence of labelled K_2p_3.1 protein (205% ± 13.5%) compared with the signal from myocytes from the subepicardial region (100% ± 5.3%) (Figure 1C; *n* = 92 cells per group; *P* < 0.0001). This significant difference of immunofluorescence was maintained when the signal was expressed relative to cell cross‐sectional area (Figure 1D; *n* = 92 cells per group; *P* < 0.0001). As such, using this method total and density of K_2p_3.1 protein expression were determined to be higher in cells from the subendocardial region compared with those from the subepicardial region.

### 3.2 A transmural gradient of K_2p_3.1 protein expression across the left ventricular free wall

**Figure 2:**
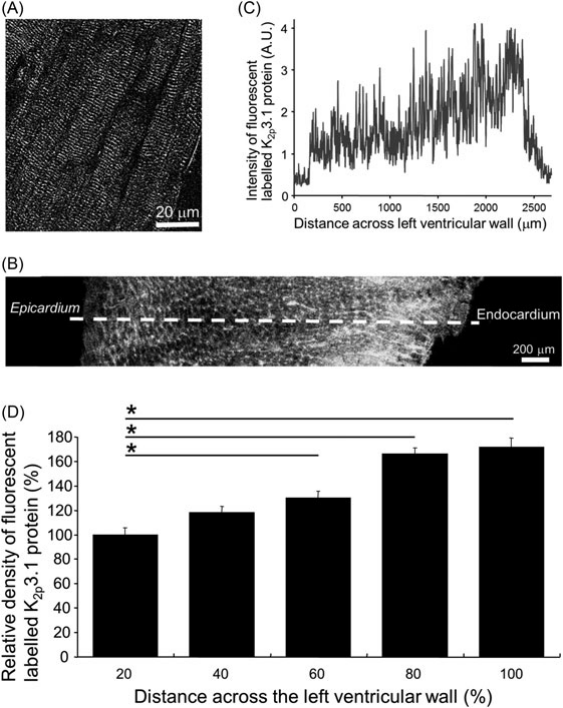
Transmural gradient of immunofluorescent‐labelled K_2p_3.1 protein expressed across the left ventricular free wall. A, Illustrative high‐resolution image of myocytes within the left ventricular wall with K_2p_3.1 protein labelled. B, Example of K_2p_3.1 protein labelling in a cross‐section of the left ventricular free wall. B‐C, A white dashed line is oriented perpendicular to the epicardial surface and is drawn to the endocardial surface in (B), which indicates the line used to determine a transmural profile of immunofluorescent labelled K_2p_3.1 protein in (C). C, A profile of fluorescence from an example tissue section shows the increase in immunofluorescent‐labelled K_2p_3.1 protein from the epicardial surface to the endocardial surface across the left ventricular free wall. D, Intensity of labelled K_2p_3.1 protein (*n* = 49) compared at 20% distance intervals across the left ventricular wall. Mean values were normalized to signal in the most epicardial interval (0%‐20% distance across the left ventricular wall). Data are shown as means ± SEM; *n* = 49 sections per group; one‐way analysis of variance with Holm‐Sidak comparisons *P < 0.001

Ventricular tissue exhibited the same pattern of labelled K_2p_3.1 protein as previously observed in myocytes with strong labelling of transverse striations across each myocyte and also present at the intercalated disc (Figure 2A). The regional distribution of immunofluorescent labelled K_2p_3.1 protein was determined across the left ventricular free wall. The profile of immunofluorescent labelled K_2p_3.1 protein intensity showed a progressive increase towards the endocardial surface. Immunofluorescence was averaged for each 20% interval distance along the profile from the epicardium for each section (Figure 2B and 2C). Significantly greater fluorescence was detected in the most endocardial segment at 80% to 100% of the transmural distance (171% ± 7.7% endocardial layer) compared with the initial epicardial 0% to 20% of the distance (100% ± 5.3% epicardial layer). Similar significant differences were noted when the 0% to 20% interval distance was compared with the 60% to 70% distance interval (130% ± 6.4%) and 80% to 90% distance interval (166% ± 8.7%) (Figure 2D) (*n* = 49 sections; one‐way analysis of variance with Holm‐Sidak comparisons *P < 0.001).

### 3.3 Quantification of K2p3.1 protein expression and mRNA in tissue isolated from subendocardial and subepicardial regions

**Figure 3:**
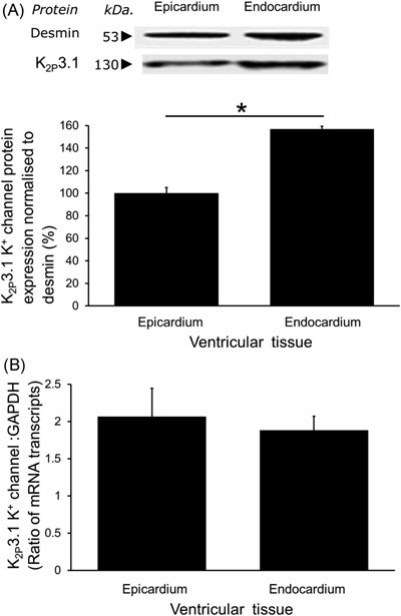
Quantification of K_2p_3.1 protein and mRNA expression. A, An example of a Western blot showing the identified specific single band labelled for K_2p_3.1 protein (and desmin protein band for normalization). Significantly more K_2p_3.1 protein was expressed in tissue from the subendocardial than the subepicardial layer (mean ± SEM; *n* = 8; Student's *t* test *P < 0.0001). B, No significant difference in the amount of K_2p_3.1 mRNA as expressed relative to GAPDH mRNA was identified between regions (mean ± SEM; n = 10). GAPDH, glyceraldehyde 3‐phosphate dehydrogenase; mRNA, messenger RNA

The expression of K_2p_3.1 protein, as assessed by Western blot analysis, was identified as a specific protein band with a molecular weight of 130 kDa in each lane of ventricular tissue (Figure 3A). We have previously shown this specific band to be absent when the anti‐K_2p_3.1 antibody is competitively inhibited with the appropriate antigenic K_2p_3.1 peptide.^10^ Furthermore, no bands were observed after omission of the primary anti‐K_2p_3.1 antibody. The integrated signal from the labelled K_2p_3.1 protein was normalized to that for labelled desmin protein as identified per tissue sample lane. Our assessment of the relative density of labelled K_2p_3.1 protein showed that tissue from the subendocardial region possessed more than 50% more K_2p_3.1 protein than tissue from the subepicardial region (157% ± 2.6% vs 100% ± 5.0%, respectively; Figure 1A; *n* = 8; *P* < 0.0001).

In contrast, analysis of K_2p_3.1 mRNA expression found there was no significant difference in mRNA content of the myocardium between the subendocardial and subepicardial layers (1.88 ± 0.2 mRNA transcript expression vs 2.06 ± 0.4, respectively; Figure 3B; *n* = 10).

### 3.4 Regional differences in K_2p_3.1 current

**Figure 4:**
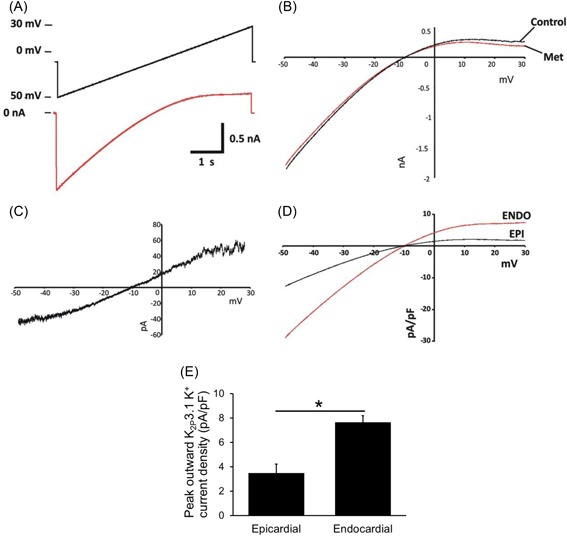
K_2p_3.1 Current‐voltage relationship in the left ventricular free wall. Subepicardial and subendocardial layer–derived myocytes were whole‐cell voltage‐clamped. A, Cells were voltage‐ramped from −50 to 30 mV over 6 seconds with a holding potential of −10 mV (upper line, black), whole‐cell currents were recorded (lower line, red). B, Current‐voltage relationship in the presence (met) and absence (control) of a specific K_2p_3.1 inhibitor methanandamide. C, Example of the methanandamide‐sensitive current‐voltage relationship from a single ventricular myocyte. D, Current‐voltage relationship from epicardial and endocardial myocytes. E, Average currents at 30 mV, taken as the peak outward current, were shown in the endocardial myocyte to be double that measured in the epicardial myocyte (Students *t* test, *P < 0.001; Epi *n* = 7; Endo *n* = 6)

We have determined a transmural gradient of protein expression but this does not automatically indicate a functional difference across the ventricular wall. Currents recorded using a slow ramp protocol (−50 to 30 mV over 6 seconds) showed a methanandamide‐sensitive component to the current‐voltage relationship (shown in Figure 4) typical of an open rectifying current with a reversal potential at −10 mV (−19.8 mV when corrected for the junction potential). Current‐voltage relationships in myocytes from both the subepicardial and subendocardial layers displayed similar rectification. Overall, a significantly larger current density was measured in myocytes from the subendocardial region (7.66 ± 0.53 pA/pF; *n* = 7) in comparison with myocytes from the subepicardial region (3.47 ± 0.74 pA/pF; *n* = 6; Figure 4E; *P < 0.001). On average the current density in myocytes from the subendocardial layer was more than double that identified in cells from the subepicardial region.

## 4 DISCUSSION

K_2p_3.1 mRNA and protein have been identified in both the atria and ventricles of the rat and human heart alike.^10^, ^20^, ^21^ In the present study we have refined this understanding confirming K_2p_3.1 expression extensively throughout the rat left ventricle, producing a significant open‐rectifying potassium current in myocytes isolated from both the subepicardial and subendocardial regions. However, K_2p_3.1 protein is expressed heterogeneously across the left ventricular wall with less than half the K_2p_3.1 current being identified in myocytes from the subepicardial region compared with that measured in myocytes from the subendocardial region. This despite a lack of differential expression of the encoding mRNA implying differences in translational regulation, trafficking or turnover of this channel. The identified heterogeneity of this channel, with a key role to play in moderating the action potential during normal physiology but particularly during pathophysiology, is of interest in determining the potential causes of arrhythmias, particularly during and post myocardial infarction.

The cardiac action potential displays characteristics that are distinct for each region of the healthy heart.^22^ The key difference in the action potential profiles across the left ventricular wall is the rate of repolarization, which occurs more rapidly in the subepicardial region of the left ventricle than the subendocardial region, principally due to heterogeneity in expression of key potassium channels.^23^ We have now identified significant differences in I_K2p3.1,_ adding to our understanding of the heterogeneity of K^+^ channel expression.^3^, ^8^ Previous measures by Putzke et al^16^ showed K_2p_3.1 to be responsible for between 3% and 40% of net outward current at 0 mV. If these measures represent the range of contributions of this channel to action potential forms in this mixed population of myocytes from across the left ventricle to repolarization the impact of the identified heterogeneity on the dispersion of repolarization is of key significance. Indeed, using a model of a subepicardial myocyte Putzke et al^16^ showed inhibition of K_2p_3.1 channels to effect a 12% to 14% prolongation of the APD. Our data now show that expression and function of this channel is actually lowest in this region with an approximate doubling of current density in cells from the subendocardial region. As such, channel modulation is predicted to have an even greater impact in myocytes from the subendocardial region. Given that myocytes from the subendocardial region already have longer APD compared with those from the subepicardial region, inhibition of K_2p_3.1 channels is therefore likely to further exacerbate this difference increasing dispersion of repolarization.

Inhibition of I_K2p3.1_ is known to promote spontaneous activity and prolong the cardiac action potential, leading to early after depolarizations (EADs) and spontaneous ectopic activity creating a substrate for ventricular arrhythmia.^8^ Factors that modulate K_2p_3.1 can arise as a result of an ischaemic insult with local accumulation of hydrogen ions being the most notable acute inhibitor under such conditions.^3^, ^19^, ^24^ The high sensitivity of K_2p_3.1 to extracellular pH in the physiological range leads to potentially complete current inhibition in the event of a regional or global ischemic insult. Even modest acidosis under low‐flow or complete ischemia will significantly reduce the current through this channel prolonging the APD and predisposing to EADs. Also associated with ischaemic insults is inflammation and the rapid activation of leukocytes that release platelet activating factor (PAF).^25^ This release of PAF in the heart is associated with APD prolongation and EADs and arrhythmias.^26^ The mechanism for this action is by inhibition of K_2p_3.1 resulting in action potential abnormalities as previously demonstrated in mouse and canine ventricular myocytes.^8^, ^19^, ^26^ We now propose this effect is compounded by the heterogeneous nature of the expression of K_2p_3.1 channels, which, therefore, is likely to create a marked increase in action potential heterogeneity during and immediately post myocardial infarction. An issue further compounded by the fact acidosis and ischemia is often itself be heterogeneous in nature with the subendocardial region commonly showing greater changes and frequency of issues than the subepicardial region.

While the rat heart differs considerably from the human in terms of size, rate and shape of the cardiac action potential, it remains one of the most widely used models used to study cardiac function, and, in particular, cardiac heterogeneity. Qualitatively, there are similarities in apparent electrical heterogeneity across the left ventricular wall between rats and humans with differences in action potential duration between the subendocardial and subepicardial region present in both species, and in each case associated with regional differences in potassium currents. A key difference between rats and humans is the duration that potassium currents act in terms of impacting repolarization. The rat action potential is quick (several to 10 seconds of msec in duration depending on heart rate) and this means only rapidly acting currents with a relatively large capacity for ion flux can significantly alter action potential duration. In contrast, the longer human action potential (100 seconds of msec in duration) gives scope for smaller and slower activating fluxes to influence action potential duration. As such, the impact of I_K2p3.1_and moderation of this component could potentially be greater in humans than the rat heart. A potential indicator of this proposed role is the fact that the volatile anesthetic sevoflurane, known to activate K_2p_3.1 channels, precipitates increased dispersion of repolarization in the human heart, an indicator of increasing action potential heterogeneity across the ventricular wall.^27^ Such an effect may be due, at least in part, to a comparable heterogeneous distribution of K_2p_3.1 channel expression in the human heart to that which we have now documented in the rat, although future analysis of human ventricular samples would be required to confirm this.

K_2p_3.1 channels have previously had limited investigation with respect to clinical issues and outcomes in the ventricle of the heart but the heterogeneity of K_2p_3.1 expression and activity has implications for understanding pharmacology of several commonly used agents. One of the most commonly used class III antiarrhythmia drugs, amiodarone has an inhibitory effect on K_2p_3.1,^28^ as do α1 adrenergic agonists.^4^ The heterogeneous impact and prolonging effect on the cardiac APD under some conditions may be antiarrhythmogenic, but the implications are that by extension of the effects seen with PAF and other blockers they could also be proarrhythmogenic. Indeed, even amiodarone has been shown to be proarrhythmic in several cases (eg, Stanton et al^29^), although its polygenic actions complicating direct interpretation of mechanism actions on K_2p_3.1 could be key. In contrast to the numerous agents that can inhibit K_2p_3.1, inhalational anesthetics such as sevoflurane and isoflurane at clinically relevant concentrations can activate human K_2p_3.1 causing significant APD shortening. As mentioned above such effects appear to have a heterogeneous impact, which fits with the current data and has the potential to contribute to arrhythmia formation.^4^, ^27^ Such impacts are, however, complex to interpret, particularly under conditions of cardiac surgery and where other interactions with local and circulating inhibitory factors are occurring.

## 5 CONCLUSION

In conclusion, a transmural gradient of K_2p_3.1 protein expression exists across the left ventricular wall with expression increasing from the epicardial to the endocardial surface. This transmural gradient of K_2p_3.1 function is likely to contribute to electrical stability of the left ventricular wall, while being highly susceptible to physiological and pharmacological modulation that can promote arrhythmogenesis.

## CONFLICTS OF INTEREST

The authors declare that there are no conflicts of interest.
